# Better Conversations Communication Partner Training for Progressive and Non‐Progressive Aphasia: What Can We Learn From Intensive Conversation Groups?

**DOI:** 10.1111/1460-6984.70296

**Published:** 2026-07-30

**Authors:** Anna Volkmer, Nina Unger, Rosemary Townsend, Firle Beckley, Mah Rana, Elisabeth Feest, Alys Hollyer, Lyrissa Dixon, Katzia Watkins, Suzanne Beeke

**Affiliations:** ^1^ Department of Language and Cognition Division of Psychology and Language Sciences UCL London UK; ^2^ Department of Neurology University Medicine Greifswald Greifswald Germany; ^3^ Dyscover Ltd. Leatherhead Surrey UK; ^4^ FirleB Therapies UK & Lemonade from Lemons Community Interest Company Eastbourne East Sussex UK; ^5^ Royal College of Art & Birkbeck University of London London UK; ^6^ University Hospitals Sussex NHS Foundation Trust, UK

**Keywords:** aphasia, communication partner training, groups, intensive, primary progressive aphasia

## Abstract

**Background:**

Better Conversations Communication Partner Training (CPT) is delivered to dyads comprising a person with a communication difficulty and a key communication partner (CP). Better Conversations focuses on individualised strategies to promote enjoyable and successful interactions. There is no research evidence about the optimal intensity or dosage for CPT. However, intensive comprehensive aphasia programmes are increasingly offered to deliver the high dose needed for effective intervention. This service improvement project aimed to explore the acceptability and outcomes from a BC Intensive Conversation Group for family dyads.

**Methods:**

We developed separate intensive group programs for non‐progressive aphasia and primary progressive aphasia (PPA). Each was delivered to four (non‐progressive aphasia) or five (PPA) family dyads in a group for 8 days over 2 weeks (4 days face‐to‐face, four remote) in a university clinic. Intervention comprised understanding how conversation works, identifying individual dyad facilitators and barriers to conversation (using video feedback), practice (in the group and across dyads) of conversation strategies and psychological support. Participants were interviewed about their experiences and acceptability of the intervention. Outcomes post‐treatment and at 3‐month follow‐up included goal attainment, conversation behaviours, communication confidence, participation and relationships. Data were analysed using descriptive statistics and content analysis.

**Results:**

Participants received a total of 26 h of Better Conversations CPT, delivered for 2.5–3 h per day, over 2 weeks (4 days a week). Participants valued the group format and preferred face‐to‐face sessions. Dosage was variably tolerated across the groups; people with PPA and their CPs reported the group was too long and intensive, while people with aphasia and their CPs reported intensity and length were acceptable. Of a total of 31 goals set, participants self‐rated as having achieved 26 goals and rated no change on five goals. Outcome measures indicated increased confidence and participation across participants with PPA and non‐progressive aphasia in both groups.

**Conclusions:**

This service improvement project demonstrates that intensive conversation groups can be acceptable to participants with PwA, PwPPA and their CPs. Participants achieved their conversation goals and reported increased confidence and participation. Future research is required to explore the benefits of different dosages and intensities of CPT and the schedule of interventions over different disease trajectories.

**WHAT THIS PAPER ADDS:**

*What is already known on the subject*

- Better Conversations Communication Partner Training (CPT) supports people with communication difficulties and their key communication partners through individualised strategies.
- There is limited evidence on the optimal dosage or schedule for CPT, although intensive aphasia programs are increasingly used to deliver high‐dose interventions.

*What this paper adds to the existing knowledge*

- This service improvement project piloted an intensive group‐based model of Better Conversations CPT for family dyads, including those with non‐progressive aphasia and primary progressive aphasia (PPA).
- Intensive conversation groups (27 h over 2 weeks) were acceptable to participants with aphasia and their communication partners, who achieved most of their conversation goals and reported increased confidence and participation.
- Participants valued the group format and preferred face‐to‐face sessions, though tolerance for intensity varied: people with PPA found the program too long and intensive, while those with aphasia found it acceptable.

*What are the potential or clinical implications of this work?*

- Intensive CPT is feasible to deliver and benefits family dyads, including those with non‐progressive aphasia and primary progressive aphasia (PPA).
- Findings highlight the need for future research on tailoring dosage and scheduling across different conditions and disease trajectories.

**Abbreviations**
BC: better conversations
CP: communication partner
CPT: communication partner training
lv: logopenic
nfv: nonfluent agrammatic
PPA: primary progressive aphasia
PwA: person with aphasia
PwPPA: person with PPA
SLT: speech and language therapists

## 1 Background

Aphasia associated with non‐progressive conditions, including stroke and brain injury, and progressive neurological diseases such as primary progressive aphasia (PPA), has a significant negative impact on conversations and relationships (Dar et al. 2025; Pozzebon et al. 2018). Families report reduced frequency and duration of conversations with individuals with aphasia, and the need to engage in delicate management of certain topics to maintain relationships (Dar et al. 2025). It is not uncommon for partners and family members to report a change in relationship dynamic and associated burden with the onset of aphasia (Dar et al. 2025; Pozzebon et al. 2018).

Importantly, the underlying disease trajectories in non‐progressive aphasia and PPA are quite different and place differing additional stressors on relationships. People with aphasia (PwA) who experience an acute onset are often described as living with chronic aphasia beyond the initial recovery period, which can result in long‐lasting dependence on significant others for communication support (Dar et al. 2025). In contrast, people with PPA (PwPPA) experience a gradual dissolution of speech and language over time (Hardy et al. 2025), which incrementally increases distance in a relationship, causing family members to feel the person with PPA is ‘disappearing’ or ‘becoming more alien’ to them (Volkmer et al. 2024). Spouses of people with PPA have described the burden on their relationships as a ‘terrible terrible journey’ (Pozzebon et al. 2017), likening the slow progression to ‘a thousand cuts’ (anonymous quote from a care partner).

PwA, PwPPA and their families have all identified conversation and relationships as a priority in their lives. Work to develop a core outcome set for stroke‐related aphasia has shown that 39 PwA and 29 family members across seven countries prioritised being able to express their emotions and opinions with family and friends (Wallace et al. 2017a). Based on the same consensus methodology used by Wallace et al. an international collaboration across 15 countries asked 82 PwPPA and 91 family members what they would most like to change about the way PPA affects their lives. Participants highlighted ‘talking together and conversations with family and friends’ (Volkmer et al. 2024). Wallace et al. (2017b), followed their work with PwA up by asking 318 clinicians and managers (speech and language therapists, psychologists and other clinicians) what the most important outcomes from aphasia treatment are. They found that ‘being able to participate in different roles and contexts’ and improving ‘family/carers skills as better communication partners’, were the highest identified constructs. This emphasises the need for clinicians and researchers to focus on interventions that address conversations and relationships.

Communication partner training (CPT) is an established intervention that supports people with communication difficulties and significant others to have better conversations (Cruice et al. 2018; Simmons‐Mackie et al. 2016). CPT can be delivered to a person with a communication difficulty, their communication partners or both together, as a dyadic intervention (Simmons‐Mackie et al. 2016; Folder et al. 2024). Systematic review evidence for the impact of CPT currently exists for PwA (Simmons‐Mackie et al. 2016; Shrubsole et al. 2023), traumatic brain injury (Behn et al. 2021) and dementia (Folder et al. 2024). Importantly, CPT is considered one of several interventions that can be protective of mental health in stroke aphasia (Baker et al. 2018). Given the significant impact that low mood, depression and anxiety have on the lives of people with dementia (Guarnera et al. 2023), it is likely that CPT is similarly protective of mental health and likely to promote independence and improved quality of life for Pw PPA.

Whilst there have been several interventions demonstrating the positive impact of CPT, none have explored the ideal dosage (total hours), intensity (spacing of hours) and schedule (frequency over time) of the intervention. A review by Cruice et al. (2018) highlights that within the 56 studies of CPT for stroke aphasia included in the Simmons‐Mackie et al. (2016) review, dosage ranged from 2 to 20 sessions, with most delivered once weekly for an hour. The authors highlight a lack of information about schedule across studies. Folder et al. (2024), in their review of 30 studies exploring CPT interventions for families of people with dementia, found great variability in dosage and intensity. CPT programs ranged from 1 to 18 sessions delivered once weekly for the most part, with an average of 1 h and 20 min per session. None of the 56 stroke aphasia and 30 dementia CPT studies included in these reviews proposes any benefits or disadvantages for CPT dosage or intensity. However, clear benefits of increased intensity have been demonstrated for intensive comprehensive aphasia programs, known as ICAPs (Monnelly et al. 2022). Monnelly et al.’s review of ICAP interventions reported that most have an impairment‐based focus and incorporate other approaches (such as CPT) as additions to achieve comprehensiveness. The question of the effectiveness of high‐intensity CPT in its own right remains unexplored.

Better Conversations (BC) is an approach to CPT informed by applied Conversation Analysis and behaviour change theory. It is delivered to dyads comprising a person with a communication difficulty and a key communication partner (CP) (Beeke & Bloch, 2022). BC focuses on the identification by dyads of individualised strategies (during goal setting) that are then practised to promote enjoyable and successful interactions. Better Conversation with Aphasia (BCA) is delivered over eight 1‐h sessions (Best et al. 2016), once weekly, whilst Better Conversations with PPA (BCPPA) is delivered over four 1‐h sessions, once weekly (Volkmer et al. 2018). Both BCA and BCPPA were originally designed to be delivered to individual dyads, and published studies have reported on this delivery modality (Best et al. 2016; Volkmer et al. 2023). Whilst the dosage for both BCA and BCPPA was chosen to promote implementation in an NHS setting (Volkmer et al. 2021), people affected by PPA and treating speech and language therapists (SLTs) in an NHS‐based pilot trial reported they would have benefitted from a higher dosage (Vokmer et al. 2022). In both the BCA (Best et al. 2016) and BCPPA studies (Volkmer et al. 2023), participant dyads recorded several 10‐min conversation samples before and after therapy, which were analysed by raters (masked to timepoint) to code and count the presence of conversation behaviours. Identified conversation behaviours were selected in line with each dyad's goals set during therapy, and conversation analysis informed definitions of observable behaviours. Statistical analysis was informed by participants’ desire to increase or decrease a behaviour. Results demonstrated improvements in conversation behaviours that aligned with goal achievement across both studies, alongside positive changes during the BCPPA study (Volkmer et al. 2023) in self‐rated quality‐of‐life measures (using the Aphasia Impact Questionnaire (AIQ; Swinburn et al. 2019). Additionally, participants with PPA and their families reported that BCPPA provided benefits in confidence, participation and relationship that were not captured by the AIQ (Volkmer et al. 2022). Moreover, coding and counting conversation behaviours is a time intensive measurement approach, which is difficult to replicate in a clinical environment. This underlines the importance of investigating both clinically meaningful constructs and clinically viable outcome measurement tools in CPT intervention research.

Current European Stroke Organisation (ESO) guidance for aphasia (Brady et al. 2025) and best practice principles for speech and language therapy for PwPPA (Brady et al. 2025) make specific recommendations for dosage and modality. Key ESO recommendations highlight that ‘people with aphasia after stroke, should have the opportunity to access speech and language therapy frequently (we suggest at least four times weekly), intensively (we suggest at least 3 h weekly)’ and that to achieve this ‘alternative approaches to therapy delivery such as digitally‐delivered SLT or group‐based SLT approaches may augment therapy provision’ (Brady et al. 2025). PPA best practice principles do not make any specific recommendations around dosage and intensity, but do refer to the schedule of interventions, emphasising the need for ongoing opportunities for review (Volkmer et al. 2023). Given that PwA have been shown to initiate more communication, across a greater range of modalities for more varied purposes in groups versus individual therapy (Fama et al. 2016), group delivery of CPT has the potential to increase dosage and provide opportunities to augment intensity.

Influenced by these guidelines, the current study reports on a service improvement project in a university clinic staffed by experienced SLTs supervising student SLTs, which piloted a BC intensive conversation group to explore the following questions:

• Is it acceptable to deliver a higher dosage and intensity of BC CPT in a group format for PwA and their CPs, and (separately) for PwPPA and their CPs?
• Will participants benefit from BC intensive conversation groups, in terms of conversation behaviours, confidence, participation and relationships?
• Will any benefits be maintained 3‐months after attending BC intensive conversation groups?

## 2 Methods

### 2.1 Design

**FIGURE 1:**
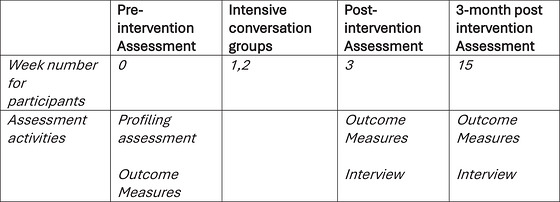
Participant flow through the study.

In this university clinic service improvement project, two intensive conversation groups delivered the BC approach to CPT for PPA, and separately for stroke aphasia, for a total of 26 h over 4 weeks during March and April 2024. The PPA group took place in the first 2 weeks, and the stroke aphasia group in the second 2 weeks, with each person participating for a total of 15 weeks from pre‐assessment to 3‐month follow‐up (see Figure 1).

All participants were provided with accessible participant information in the form of a video recording alongside written and pictorial information sheets. Consent processes were undertaken by trained SLTs, who are skilled in providing all practicable supports to people with communication difficulties. For the purposes of this study, these included short sentences, repetition, written and visual information and verbal explanations of technical terms. The consent process was supported by student SLTs. With these supports, all participants were able to provide informed written consent to participate.

### 2.2 Recruitment

Participants were purposefully recruited from referrals to the UCL Communication Clinic (https://www.ucl.ac.uk/brain‐sciences/pals/research/language‐and‐cognition/ucl‐communication‐clinic) and via the PPA support group membership of Rare Dementia Support (https://www.raredementiasupport.org/). We aimed to recruit four or five dyads per group. Potential participants were identified by the manager of the UCL Communication Clinic and the direct support team at Rare Dementia Support (a UK third sector organisation), based on their knowledge that PwA or PwPPA had not previously received CPT and/or had expressed an interest in it. The lead author, A.V., contacted potential participants via email or telephone to invite them to attend. Interested participants were screened on a first come, first served basis, based on both information in their referral and questions asked on contacting them, against inclusion criteria: a relevant diagnosis (non‐progressive aphasia or PPA); a CP able to attend all sessions; able to travel to in‐person sessions in central London; access to a device (tablet or computer) and the internet to attend online sessions via Zoom. Participants were excluded from the study if they had multiple diagnoses that could impact their communication, for example, stroke and dementia, or a serious mental health diagnosis. CPs were excluded from the study if they had a neurological diagnosis that could impact communication and participation, for example, dementia. Whilst participants were not excluded based on PPA variant or aphasia severity, they were required to have adequate communication skills to participate in group activities (as judged by the first author on screening).

Having agreed in principle to participate in the group, an email was sent with a link to video‐recorded information regarding the details and commitment to the group. Participants were offered reimbursement for all travel, plus accommodation costs if not living in London. Refreshments were provided for in‐person sessions.

### 2.3 Acceptability

Participants gave their opinions on the intensive conversation groups in a post‐intervention semi‐structured dyadic online interview held on the video conferencing platform Zoom. Interview questions were developed by A.H., E.F., A.V. and S.B., targeting perceived gains, acceptability of dosage and modality (group, online and in person) and group activities (see Appendix 1). Immediately post‐intervention, two student SLTs involved in group delivery (A.H. and E.F.) conducted the interviews (one student per dyad). Whilst this ensured participant dyads were at ease with known interviewers, there was potential for bias. Therefore, a repeated opportunity for feedback was provided at a 3‐month follow‐up meeting with student SLTs L.D. and K.W., who were not previously known to participants. Having two opportunities for feedback was also considered useful to ensure participants had adequate time to reflect on the impact of the intervention, and any recommendations for change. Student SLTs used supported communication strategies such as repeating questions, providing a written version (e.g. on a slide) and explaining what a question meant. All semi‐structured interviews were video recorded and automatically transcribed via Zoom. Transcripts were checked and corrected by A.V. and anonymised by A.V. and N.U.

### 2.4 Assessment and Outcome Measures

Measures were administered online (on Zoom—with all participants attending on personal devices from home) by student SLTs in the week prior to the intensive conversation groups commencing. These were repeated in the week following group attendance (A.H., E.F.), and at 3‐month follow‐up (L.D., K.W.), supervised by A.V. and S.B. While initial and immediate post‐intervention measures were administered by student SLTs involved in delivering the intensive conversation groups, 3‐month follow‐up measures were administered by student SLTs not known to participants. Measures were selected either to profile language, and thus only completed prior to therapy, or as outcome measures (see Table 1).

**TABLE 1:** Assessment.

**Profiling assessment tools**	**Who was assessed?**
Sentence comprehension and picture description task from the Comprehensive Aphasia Test (CAT; Howard et al. 2010)	*PwA/PwPPA*
Video recorded conversation sample—analysed using the Better Conversations checklist	PwA/PwPPA and their CPs
**Outcome measurement instruments**	**Who was measured?**
Video recorded conversation sample— Kagan Scales: Measure of Skill in Supported Conversation (MSC) and Measure of Participation in Conversation (MPC) (Mok et al. 2021)	PwA/PwPPA and their CPs
Goal Achievement	PwA/PwPPA and their CPs
Communication Confidence Rating Scale for Aphasia (CCRSA) (Babbitt et al. 2011)	PwA/PwPPA
Communication Participation Item Bank (CPIB) (Baylor et al. 2013)	PwA/PwPPA
Quality of Caregiver Patient Relationship (QoCPR; Spruytte et al. 2002)	PwA/PwPPA and their CPs

Conversation samples were collected for two purposes: to inform intervention video feedback and as an outcome measurement via use of the Kagan Scales (Mok et al. 2021). Dyads were recorded over Zoom at three time points, having a 10‐min conversation on a topic of their choosing, having been advised that topics around planning future events such as holidays were recommended in coproduced guidance from the BCPPA intervention manual (Volkmer et al. 2021). All dyads sat together in the PwA/PwPPA's home, sharing the same camera, in a room of their choosing. Participants were given verbal guidance not to look at the camera, but to face one another during the recording. The assessor turned off their camera and microphone and left the room during the recording. To inform intervention delivery, the recordings were analysed using the Better Conversations Facilitators and Barriers Observation Checklist (Beeke and Bloch 2023) prior to the groups starting, to select relevant clips (two or three clips of approximately 30–45 s duration) to use for video feedback with individual dyads during intervention. The Kagan Scales (Mok et al. 2021) were used as an outcome measure for each member of the dyad. Both the stroke aphasia and dementia versions of the Kagan Scales were explored a priori and, given the pattern of behaviours observed in prior PPA studies (Volkmer et al. 2023), the Kagan Scales for aphasia were selected for use with both groups based on their increased relevance for PPA.

Other outcome measures used included the Communication Confidence Rating Scale for Aphasia (CCRSA; Babbitt et al. 2011), Communication Participation Item Bank (CPIB; Baylor et al. 2013), Quality of Caregiver Patient Relationship (QoCPR; Spruytte et al. 2002) and goal achievement. Goal setting is an integral aspect of the BC intervention. Supported by the SLT, participants set goals on Day 1 (see Table 2) about what they would like to work on, after having viewed the video recording of their own conversations. Using our goal achievement measure (influenced by Goal Attainment Scaling; Turner‐Stokes 2009), participants are then asked to rate the importance (as important, or less important) and achievability (more, or less, achievable) of their goals. At the end of the intervention period (on Day 8—see Table 2), participants are asked to rate their goals as 2: I am doing the goal a lot more; 1: I am doing the goal a bit more; 0: no change. Table 2 provides an overview of profiling assessments and outcome measures and who completed them, see Appendix 2 for a more detailed description of the assessment tools.

**TABLE 2:** Dosage and intensity of Better Conversations Intensive Conversation Group sessions.

Week 1				
Day 1—*In person* *Dosage*—*5 h*	10:30–11:00 Introductions	11:00–12:00 Discussion about how conversation works	12:00–13:00 Group lunch	13:00–15:30 Video feedback and goal setting
Day 2—*In person* *Dosage*—*4 h*	10:30–12:00 Sharing of goals		12:00–13:00 Group lunch	13:00–14:30 Dialogic art activity—facilitated by artist‐researcher (M.R.)
Day 3—*Online* *Dosage*—*3 h*	10:30–11:00 Review of home practice	11:00–12:00 Creative activity—SLT collaborator (F.B.)	Break from computer	13:00–14:30 Rotating pairs practice
Day 4—*Online* *Dosage*—*3 h*	10:30–11:00 Review of home practice	11:00–12:00 Rotating pairs practice	Break from computer	13:00–14:30 Group conversation

Week 2				
Day 5—*Online* *Dosage*—*2.5 h for CP, 1.5 for PwA/PwPPA*	10:30–11:00 Review of home practice	11:00–12:00 Problem solving	Break from computer	13:00–14:00 Counselling with partners only
Day 6—*Online* *Dosage*—*2.5 h for PwA/PwPPA, 1.5 for CP*	10:30–11:00 Review of home practice	11:00–12:00 Rotating pairs practice	Break from computer	13:00–14:00 Supported conversation: PwA/PwPPA only
Day 7—*In person* *Dosage*—*3.5 h*	10:30–11:00 Review of home practice	11:00–12:00 Discussion about sharing strategies with other people	12:00–13:00 Group lunch	13:00–14:00 Guided walk
Day 8—*In person* *Dosage*—*3.5 h*	10:30–11:30 Review of goals	11:30–12:00 Creative activity—Rocking Aphasia	12:00–14:00 Group conversation in restaurant	

*Note*: Total dosage = 26 h.

### 2.5 Intervention Procedures

Dyads attended an 8‐day CPT intensive conversation group over a period of 2 weeks, 4 days per week, 4 h each day. Days 1 and 2, and 7 and 8 were delivered face‐to‐face in a university clinic. All other days were delivered as synchronous telehealth sessions via video conferencing (using Zoom). Table 1 provides details of session length and intensity. The two groups were consecutive, a PPA group followed by a non‐progressive aphasia group and delivered by two student SLTs (A.H. and E.F.), four qualified SLTs (A.V., S.B., R.T. and F.B.) and an artist‐researcher (M.R.).

The CPT intensive conversation groups were designed by A.V. and S.B., with advice from PR, a PPI advisor for the BC research group and CP of a PwPPA. The CPT intensive conversation groups delivered the core components of the BC approach to CPT, namely reflecting on how conversations work, video feedback to support identification of barriers and facilitators in a dyad's own conversations, goal setting, and practice of chosen conversation strategies (see Beeke and Bloch 2023). Dyads reviewed their conversation videos in individual sessions with student SLTs A.H. and E.F., supported by A.V. and S.B., whilst remaining dyads did group activities. Based on these video observations, both members of each dyad set goals to increase facilitators (strategies) in conversation and reduce barriers. After all dyads had completed an individual video reflection and goal setting session, all participants shared and finalised their goals within the group. Opportunities to practise strategies were embedded throughout the intensive conversation groups through activities such as individual dyad practice conversations with other group members giving feedback, CP swapping, group conversations in increasingly larger numbers, and in the community, such as having a meal in a restaurant. Participants also explored how to share their learning and strategies with their social networks. Naturalistic opportunities for conversation practice were provided during refreshment breaks, a guided walk and dialogic group art activities. Home practice was encouraged throughout to promote carryover of target strategies. At the suggestion of PR, our PPI co‐author, CP were given an opportunity to meet separately from their partners to participate in peer support and counselling. Table 2 provides the dosage and intensity of intervention sessions. A completed Template for Intervention Description and Replication (TIDieR) checklist can be found in Appendix 3.

### 2.6 Inter‐Rater Reliability

Rating of conversations using the Kagan Scales (Mok et al. 2021) was conducted by student SLTs L.D. and K.W., who were randomly allocated recordings using an online randomisation tool (https://wheelofnames.com/). Importantly, the students were also masked to the time point at which the video was made. Prior to scoring all videos, L.D. and K.W. participated in a training session where they scored one video recording. They then independently scored a further 10% of all recordings (comprising 3/26 conversation videos) and met with A.V. to discuss and agree on any discrepancies. L.D. and K.W. then continued to rate the entire sample. Inter‐rater reliability was initially calculated using percentage agreement as 53.3% for the Kagan Scales. Discrepancies were discussed, and the lead researcher, A.V., was contacted to resolve any outstanding concerns. Low reliability for the Kagan Scales has been described in the research literature (Behn et al. 2025) and was accounted for in this study by errors in scoring methods. As per Behn et al. (2025) findings, after further training, including discussion with A.V. and re‐rating of the initial training videos, L.D.’s and K.W.’s rating accuracy increased to close to 99.7%. Importantly, the 99.7% inter‐rater reliability achieved following training does not address the low reliability of the Kagan Scales and should not be misinterpreted as an assessment of the psychometric properties of the scale, but rather as informative for the reader.

### 2.7 Analysis

Recruitment and attendance data were explored by collating information about reasons for exclusion from the study and monitoring attendance.

Semi‐structured interviews were analysed using conventional content analysis (Hseih et al. 2005). This approach was felt to be most relevant given the brevity of the interviews, and the focus on capturing patterns of meaning. Given the aim of exploring intervention acceptability, interview data from immediately post‐group intervention and 3‐month follow‐up were analysed together to capture patterns of meaning. Content analysis allows for data to be summarised without immersion or exploration of latent meanings (Kleinheksel et al. 2020). A German speech and language therapy researcher specialising in dementia and independent of the therapy team N.U. supported the analytic process by providing an unbiased lens to maximise reflexivity. A.V. and N.U. independently familiarised themselves with the data through repeated reading and identification of interesting or important points. They then met to compare observations and check for inconsistencies, discussing and grouping the data in line with patterns of meaning. The identified groupings were re‐examined to check for overlap. Final theme and subtheme titles were agreed prior to the construction of a written analysis, where identical, similar and unique themes across the PPA and non‐progressive aphasia groups were colour coded and illustrated with appropriate quotes. Member checking was not used as it was felt the time that had elapsed between participants attending the group and data analysis was too great.

Individual participant Kagan Scale data was visualised using graphs, while group data was further explored using descriptive statistics such as means, ranges and standard deviations. Group data are analysed across the three measurement time points using the non‐parametric Friedman test, which is suitable for non‐normal ordinal data. For significant results a non‐parametric pairwise comparisons (Wilcoxon signed‐ranked test) between pre‐post and pre‐follow‐up to determine the location of the significant change was conducted. The Friedman analysis has a minimum recommended sample size of five, meaning the non‐progressive aphasia group was underpowered with only four participants. Therefore, the Minimal Detectable Change (MDC_90_), defined as the minimal score change that falls outside measurement error with 90% confidence, was also used to identify changes in individual participants' confidence, as measured by the CCRSA (≥ 5.96), consistent with methodology applied by Dignam et al. (2025). Similarly, the MDC_90_ for the CPIB (≥4.61), as applied by Carlozzi et al. (2021), was used to identify changes in individual participants’ participation. There is currently no MDC_90_ data available for the Kagan scales or the QoCPR measure.

## 3 Results

### 3.1 Participant Recruitment and Retention

Table 3 provides an overview of participant demographics and pre‐intervention language profiling results. Of the five potential participant dyads contacted to invite them to a PPA group, all consented to participate. Of the 11 potential participant dyads invited to the non‐progressive aphasia group, four consented to participate. Of the seven who declined, four potential participants with stroke aphasia did not have a CP who was able to commit to attend the group, and three did not feel the group was of interest or benefit to them.

**TABLE 3:** Participant demographics and pre‐intervention language profiling results.

	**Age**		**Gender**		**Languages spoken**		**Occupational background**		**Relationship**	**PPA variant**	**CAT—sentence comprehension scores**	**CAT—picture description scores**	**Time since symptom onset**	**Time since diagnosis**
**PPA group**	PwPPA	CP	PwPPA	CP	PwPPA	CP	PwPPA	CP						
PPA dyad 1.01	77	59	M	F	American English	British English/ Cantonese	Government employee	Research chemist	Partners	nfv	23/32	47	7 years, 3 months	6 years, 7 months
PPA dyad 1.02	55	53	F	M	British English	British English	IT Project Manager	IT Project Manager—still working FT	Spouses	Lv	20/32	24	1 year, 2 months	9 months
PPA dyad 1.03	80	70	M	F	British English	British English	Director and manager	PA	Spouses	nfv	18/32	59	2 years, 7 months	2 years, 1 month
PPA dyad 1.04	77	82	F	M	British English	British English	Director of literature at Arts Council	Architect	Spouses	Lv	25/32	83	7 years, 6 months	3 years
PPA dyad 1.05	84	82	M	F	British English	British English	Journalist	Journalist	Spouses	Lv	29/32	53	8 years	2 years, 2 months

Aphasia group	Age		Gender		Languages spoken		Occupational background		Relationship		CAT—sentence comprehension scores	CAT—picture description scores	Time post stroke	
	PwA	CP	PwA	CP	PwA	CP	PwA	CP						
PwA dyad 2.01	72	67	M	F	British English	British English	Electrical engineer	Administrator	Partners		30/32	7	5 years	
PwA dyad 2.02	72	59	F	M	British English	British English	Record shop owner	Counsellor	Partners		16/32	14	2 years, 3 months	
PwA dyad 2.03	43	38	M	F	French/British English	British English/French	IT implementation specialist	Stay at home mum	Partners		25/32	18.5	1 year, 8 months	
PwA dyad 2.04	73	61	M	F	British English	British English	Pilot	OT	Partners (since the stroke)		24/32	0	2 years, 11 months	

*Notes*: Participants have been assigned numbers to maintain anonymity with PwPPA assigned a number commencing ‘1’ and PwA assigned a number commencing ‘2’.

Abbreviations: CAT, Comprehensive Aphasia Test (Howard et al. 2011); CP, communication partner; F, female; lv, logopenic; M, make; nfv, nonfluent agrammatic; PPA, primary progressive aphasia; PwA, person with aphasia; PwPPA, Person with PPA.

For both groups, all participants attended 100% of sessions. One dyad in the non‐progressive aphasia group did not respond to emails or telephone calls to organise the final 3‐month follow‐up meeting. One CP of a person with non‐progressive aphasia did not attend the post‐intervention assessment session.

### 3.2 Acceptability

There were many similarities in identified themes across both the PPA and the aphasia group interview data. In total, 14 themes were identified across all the interview data. A total of four themes were almost identical across the PPA and aphasia groups, two of these were identically concerned with group work, whilst two others were focused on matching group members in terms of severity and/or stage. A total of eight themes had some similarities across the PPA and aphasia groups and revealed that participants felt the groups were worthwhile, flagged a preference for face‐to‐face over online attendance, and expressed opinions on the total length of the group and a general desire for more therapy. Two completely unique themes were identified; one theme reflected that participants with PPA and their CPs identified the goal setting as a vital component of their experience, whilst another theme highlighted that for participants with aphasia and their CPs the inclusion of the CPs was a new experience and important component. Table 4 provides an overview of all themes with illustrative quotes.

**TABLE 4:** Themes identified in acceptability interviews for PPA and aphasia intensive conversation groups with illustrative quotes.

**PPA group themes**			**Aphasia group themes**	
**Main themes**	**Supporting quotes with dyad numbers**		**Main themes**	**Supporting quotes with dyad numbers**
1. It was a worthwhile investment—a preventative proactive medicine	‘We started to get a little bit of insular type stuff going on which is not good. Oh, as far as I'm concerned, it wasn't good for us. But now I think we're planning things—which is nice.’ [CP 1.03] ‘It's brought more to the forefront. So in my mind. The things we need to think about and do rather than being reactive and make changes. I think, perhaps would be more proactive.’ [PwPPA 1.05] ‘Being able to now have more confidence in saying something.’ [CP 1.01]		1. You have nothing to lose and plenty to gain	‘Nothing easy is worth having anything you get as a result of hard work you wanna hold on to.’ [CP 2.02] ‘I think that is, that is a confidence thing. I think that's what the conversation group did. It boosted your confidence to try and get involved more.’ [CP 2.03] ‘His speaking obviously, has improved.’ [CP 2.03]
2. Working in a group was really valued	‘It was so good to be able to talk to other people in the same situation, who understand what it's about.’ [CP 1.05] ‘Everyone's energy levels are gonna vary through the day. And you could see that in the room, and I think if it's just you with a therapist yeah. Or if it's a group. it might be a period where one part of the room is a bit more lively, and you're sitting back a bit back. and that's fine.’ [CP 1.02] ‘I think at home we probably wouldn't try them out. We might think it's silly. He might think it's silly, but because we're with you guys. And you kept saying, do gestures do gestures. It was part of the whole group.’ {CP 1.01] Meeting people with the similar problems and the ability to find a gold nugget in the interaction [PwPPA 1.05] ‘Your embarrassment or awkwardness goes away because everyone else is in the same boat.’ [CP 1.02]		2. It was good to be part of a group—it felt like a safe space	‘It was the first time I had met anyone else like that.’ [CP 2.03] ‘It's the first time that I'm aware of that PwA has met other people similar difficulties to herself. I felt that that would have helped to not feel so alone. You know there's other people with similar struggles.’ [CP 2.02]
3. The goals were the key—the strategies	I think learning from other people is really important. Especially around setting goals [CP 1.01] ‘The first appreciation I have is for the SLT so she ran a feedback session where we concluded with my—our goals.’ [PwPPA 1.03]		3. It needed to be that long to achieve what we did	‘It needed to be that long, because I think if it was shorter, I don't think it would have been as effective.’ [CP 2.03] ‘I found it tiring but beneficial at the same time. The facts are that it pushed me. In a positive way.’ [CP 2.02]
4. The group did not need to be so long—it was tiring for PwPPA and CPs	‘I think it was just because we're not used to doing that. And you know, to sit here all day as well. I just absolutely think, Hallelujah, I don't go to work anymore and have to sit in front of the screen all day, but, you know, we're 80. So what do you expect? You know we're gonna feel it? Is it? Was it our the age thing, or was it just that we're not used to it? But I think it maybe is. It could be trimmed slightly.’ [CP 1.03] I think it's would be useful to have it a day less [PwPPA 1.03]		4. Face‐to‐face delivery was just more effective	‘I reckon in person. Yeah.’ [PwA 2.03] ‘Natural questions happening in lunch and break time, and that just gave people the opportunity to practice what we had been doing in the session before’ [CP 2.03] ‘I don't think you get that sense of community. If it was solely online, I don't think that would have been there’ [CP 2.02]
5. We preferred face‐to‐face—did we need to do it online?	‘Zoom is just more one dimensional. I think you don't have that same—It's difficult to replicate that sense of being in a group and interacting as a group.’ [CP 1.02] Because of the difficulty of getting the app and just understanding how it worked. got very tedious, because that took a long time. And then what we put up photographs of places that meant something to us, and asked everybody else to scribble over them. and that didn't feel appropriate [PwPPA 1.05] ‘The first zoom day was crippling.’ [CP 1.03] That's there's going to be more of the travel issue, and that's the advantage of Zoom. You can offer that. Offer it to people wherever they are, whatever their circumstances. [CP 1.05]		5. Matching group members would help challenge us to improve	‘I just don't know if that would be a way to plan it like in terms of matching people.’ [CP 2.03] ‘Cause I feel like some at some point, I wonder if he would have been pushed a bit more to try more things if there was somebody who was that? Iin a similar situation in terms of, you know, could put some sentences together, and you could have more back and forth.’ [CP 2.03]
6. Matching group members to stage and PPA type might be useful	‘Mixing similar stages—so you wouldn't want to mix another [different stages of PPA] Yeah, they wouldn't. They'd find it difficult.’ [PwPPA 1.05] ‘1.04 was quite, I thought one of the better. most articulative of the of our group. Didn't you think. I didn't feel particularly in it.’ [CP 1.04]		6. It is the first time I was involved as a CP	‘Interesting to meet other people and partners and different people's experience—I've never met anyone else.’ [CP 2.03] ‘It's the first time that I've done something with you.’ [CP 2.03] ‘The feel that I'm part of a a new community. To realize that there, there is help out there…that that I'm not alone anymore.’ [CP 2.02]
7. We need more follow‐up opportunities afterwards	‘We enjoy continuing to meet up with (names) after the group.’ [PwPPA 1.01] ‘When we met to do a talk together that was important.’ [PwPPA 1.05]		7. We need more therapy after this	‘It would be useful to catch up with people from the previous group as another one off get together.’ [CP 2.01] ‘We need more ongoing support for his word finding.’ [CP 2.03]

*Note*: Green represents themes that are the same across groups. Orange represents themes that have similarities and white represents different themes.

### 3.3 Conversation Outcomes

Conversation outcomes were captured via our goal achievement measure and evaluation of conversation samples using the Kagan Scales. On Day 1 of the intensive groups all participants in the PPA group set individual conversation goals, and 7 of 8 participants in the non‐progressive aphasia group (one CP was unable to identify a personal goal, despite support). At the end of the intensive conversation group (Day 8), of the 18 goals set by participants in the PPA group, five were rated as 2 (*I am doing the goal a lot more)*, eight as 1 (*I am doing the goal a bit more*) and five as 0 (no *change*). In the PPA group One dyad achieved one goal of the three they set; three dyads achieved three of the four goals they set, and one dyad set and achieved three goals. Of the 13 goals set by participants in the non‐progressive aphasia group, nine were rated as 2 (*I am doing the goal a lot more)*, four as 1 (*I am doing the goal a bit more*). in the non‐progressive aphasia group One dyad set and achieved two goals, one dyad set and achieved three goals and one dyad set and achieved four goals. See Table 5 for a detailed dyad‐by‐dyad overview of goal achievement.

**TABLE 5:** Goal achievement.

PPA group goal achievement			Aphasia group goal achievement		
	Goals	Self‐rating of achievement		Goals​	Self‐rating of achievement
1.01	‐ Instead of asking general questions, use multiple choices or visual tools (F; CP)* ‐ Use a lot more facial expressions and gestures (be more Italian!) when I can't find the words (F; PwPPA) ‐ Use more short words and phrases (F; PwPPA)	2 2 2	2.01	‐ Use gesture, writing and/or my phone to support my use of words.​ (F; PwA) ‐ Use a sign to show CP when I want her to wait.​ (F; PwA) ‐ Use a sign to show CP when I want her to guess.​ (F; PwA) ‐ Practise words that CP guesses.​ (F; PwA & CP)*	2 2 2 2
1.02	‐ To do more co‐driving by giving time (F; CP)* ‐ Co‐using facial expressions (F; PwPPA & CP) ‐ To use fewer questions and make them secondary to being together (B; CP) ‐ To feel more confident talking to other people (F; PwPPA)	1 1 0 1	2.02	‐ I will use gesture to show people I want to say something. ​(F; PwA)* ‐ I will write the first word down to start a conversation. (F; PwA) ‐ To have something in common to share conversation about. ​(F; CP)* ‐ To use shared gestures—we both know what they mean. (F; CP & PwA)	1 2 2 1
1.03	‐ Take 5 min out of each day to sit down and have ‘cocktail chat’ (F; PwPPA & CP)* ‐ To use signposting to be more specific and give PwPPA time to prep (F;CP) ‐ To use facial expressions more to convey thoughts (F; PwPPA) ‐ To ask a range of questions so that PWPPA is able to elaborate on his answers e.g. Open or opinion questions (F; CP)	2 1 1 0	2.03​	‐ To express myself even if it's not perfect​ (F; PwA)* ‐ Be more confident to express myself (on deeper and more complex topics)​ (F; PwA) ‐ Could not identify a goal for herself​ (?—CP)	2 2 ‐
1.04	‐ To keep the conversation flowing by describing something so CP can guess (F; PwPPA) ‐ To stay together when we go to galleries so we can discuss the art we like and why (F; CP & PwPPA) ‐ To feel more comfortable talking to other people by being more confident (F; PwPPA)* ‐ To not give up on conversation when something goes wrong (B; PWA)	1 0 1 1	2.04	‐ We will use gestures together and the CP will clarify their meaning.​ (F; PwA) ‐ I will use facial expressions and tone of voice to show what I mean.​ (F; PwA) ‐ I will prompt PwA with the first part of a word when he gets stuck. (F; CP)​*	1 1 2
1.05	‐ To use more open questions to give PwPPA choice and confidence to answer (F; CP)* ‐ To use the phrase ‘start again’ when I am bogged down during talking (F; PwPPA) ‐ To use my word lasso to get to the correct word (F; PwPPA)	0 2 0			

*Notes*: * Most important; 2: I am doing the goal a lot more; 1: I am doing the goal a bit more; 0: no change.

Abbreviations: B, Barrier focused goal; CP, communication partner; F, facilitator focused goal; PPA, primary progressive aphasia; PwA, person with aphasia; PwPPA, person with PPA.

**FIGURE 2:**
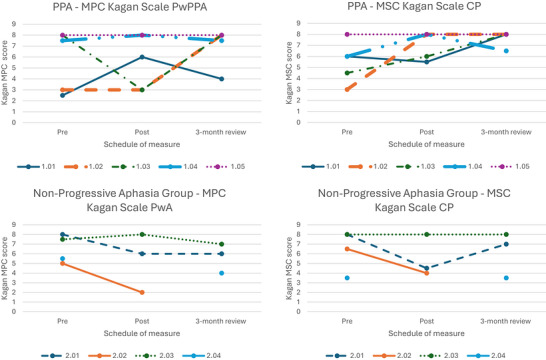
MPC and MSC Kagan Scales for each participant at pre, post and 3‐months post‐group attendance. *NB: There is missing data for two PwA and their CPs as they were unable to complete these measures at these time points*. CP, communication partner; MPC, measure of participation in conversation; MSC, measure of skill in supported conversation; PPA, primary progressive aphasia; PwPPA, person with PPA; PwA, person with aphasia.

Figure 2 demonstrates the stability or trajectory of change in conversation participation (for participants with PPA or non‐progressive aphasia) and conversation support (for CPs) measured using the Kagan Scales over three time points for each individual participant. Two of five participants with PPA demonstrated improved conversation participation on the MPC. PwPPA 1.01 improved immediately after intervention, and PwPPA 1.02 improved at 3‐month review. The scores for two participants with PPA (1.04 and 1.05) remained stable and did not change in the time between pre‐intervention and 3‐month review, however, their scores were at or near ceiling at each timepoint. One, PwPPA 1.03, showed a dip immediately after intervention but returned to their pre‐intervention level at 3‐month review. Two of the CPs in this group demonstrated an upward trajectory of conversational support on the MSC (CPs 1.02 and 1.03) although 1.02 reached ceiling immediately after intervention (maintaining a score of 8 from post‐intervention to 3‐month review). Scores for 1.01 and 1.04 increased from pre‐intervention to 3‐month review but dipped and hit ceiling, respectively, immediately after intervention. CP 1.05 scored at ceiling throughout.

Three of four participants with non‐progressive aphasia demonstrated reduced levels of participation on the MPC at the 3‐month review timepoint compared with pre‐intervention (2.01, 2.03, 2.04), however, PwA 2.03's score increased to ceiling immediately after intervention. There is missing data for PwA 2.04 at the immediate post‐intervention timepoint. There is missing data for PwA 2.02 at 3‐month review, but their score decreases immediately after intervention. Two CPs in this group (2.01 and 2.02) demonstrated reduced levels of conversational support immediately after intervention, however, the score for CP 2.01 returned to near‐pre‐intervention level at 3‐month review. There was missing data for CP 2.02 at 3‐month review. CP 2.03 remained stable at ceiling throughout. CP 2.04 had missing data immediately post‐intervention, but 3‐month review remained stable, showing no change from pre‐intervention levels.

### 3.4 Confidence, Participation and Relationship Outcomes

#### 3.4.1 Group Outcomes

Table 6 shows descriptive and statistical outcomes at a group level for confidence, participation and relationship measures. For the PPA intensive conversation group, mean confidence scores (as measured using the CCRSA, Babbitt et al. 2011) increased immediately after intervention but declined to below pre‐intervention levels at 3‐month review. In contrast, mean confidence scores for the non‐progressive aphasia group increased immediately after intervention and continued to show increased confidence at 3‐month review. For the PPA group, participation, as measured by the CPIB (Baylor et al. 2013) did not improve until 3‐month review, whilst participation increased immediately after intervention for the non‐progressive aphasia group and remained high at 3‐month review. The relationship between the dyad, measured using the QoCPR (Spruytte et al. 2002), showed no change for PwPPA or PwA but increased across both CP groups. Friedman's test showed a statistically significant difference for the CCRSA for the non‐progressive aphasia group *χ* ^2^(2) = 6.00, *p* = 0.050). Post‐hoc pairwise Wilcoxon signed‐ranked tests with Holm correction did not reveal any statistically significant difference between specific pairs of time points (all *p's* = 0.75) (note that Friedman's test requires data from each time point; thus, 2.02 was omitted due to missing data). Statistical analysis was non‐significant for all other measures, therefore no post‐hoc analysis was undertaken.

**TABLE 6:** Group level descriptive and statistical outcomes for measures of confidence, participation and relationships.

Outcome measurement tool	Who completed the measure?	PPA Group mean scores (range + Standard Deviation)				Aphasia Group mean scores (range + Standard Deviation)			
		Pre‐group	Post‐group	3‐month review	*p* value	Pre‐group	Post‐group	3‐month review	*p* value
Kagan Scales	PwPPA/PwA	5.8 (2.5–8, SD 2.79)	5.6 (3–8, SD 2.50)	7.1 (4–8, SD 1.74)	*χ* ^2^(2) = 1.07, *p* = 0.584	6.5 (5–8, SD 0.35)	5.3 (2–8, SD 1.41)	5.7 (4–7, SD 0.70)	*χ* ^2^(2) = 2, *p* = 0.368
	CP	5.5 (3–8, SD 1.81)	7.1 (5.5–8, SD 1.24)	7.7 (6.5–8, SD 0.67)	*χ* ^2^(2) = 4.13, *p* = 0.126	6.5 (3.5–8, SD 0.00)	5.5 (4–8, SD 2.47)	6.2 (3.5–8, SD 0.70)	*χ* ^2^(2) = 2, *p* = 0.368
CCRSA	PwPPA/PwA	604 (480–750, SD 101.1)	622 (505–720, SD 100.0)	542 (480–690, SD 121.9)	*χ* ^2^(2) = 3.89, *p* = 0.143	488 (320–740, SD 192)	501 (340–705, SD 164.4)	543.3 (450–710, SD 144.7)	*χ* ^2^(2) = 6.00, *p* = 0.050*
CPIB	PwPPA/PwA	8.8 (5–10, SD 3.27)	8.6 (5–18, SD 3.78)	10.6 (7–19 SD 5.12)	*χ* ^2^(2) = 5.33, *p* = 0.069	7 (0–18, SD 10.39)	8.5 (2–15), SD 7.50	8.3 (3–13, SD 5.03)	*χ* ^2^(2) = 0.67, *p* = 0.717
QoCPR	PwPPA/PwA	60.6 (55–64, SD 3.78)	60.2 (51–67, SD 6.30	—	*χ* ^2^(1) = 0, *p* = 1.0	58.3 (54–70, SD 7.84)	56.3 (51–67, SD 7.54)	—	*χ* ^2^(1) = 1, *p* = 0.317
	CP	59 (48–69, SD 6.35)	51 (44–62, SD 9.45)	—	*χ* ^2^(1) = 0.33, *p* = 0.564	56.8 (51–70, SD 8.88)	57.5 (51–70, SD 8.73)	—	*χ* ^2^(1) = 0.33, *p* = 0.564

*Note*: ^*^ significant statistical outcome.

Abbreviations: BCA, better conversations with aphasia; BCPPA, better conversations with primary progressive aphasia; CAT, Comprehensive Aphasia Test; CCRSA, Communication Confidence Rating Scale in Aphasia; CP, communication partner; CPIB, Communication Participation Item Bank; PwA, person with aphasia; PwPPA, person with primary progressive aphasia; QoCPR, Quality of Caregiver Patient Relationship.

#### 3.4.2 Individual Participant Outcomes

Of the PwPPA, two participants demonstrated gains in confidence greater than MDC_90_ on the CCRSA and two others remained stable immediately post‐group. When compared to the pre‐group timepoint, three PwPPA demonstrated a negative change greater than the MDC_90_ at 3‐months post group, though one person with PPA remained stable. Only one person with PPA demonstrated a negative change greater than MDC_90_ immediately post group, and remained stable at follow‐up. In relation to participation, all the PwPPA were stable post group on the CPIB, and one participant demonstrated a gain greater than the MDC_90_ at 3‐month follow‐up, but none declined (see Appendix 4 for a full list of individual scores).

Of the PwA, none demonstrated gains or negative change greater than the MDC_90_ on the CCRSA immediately post group. At follow‐up, however, three of the four PwA demonstrated gains greater than the MDC_90._ On the CPIB, one person with aphasia made a gain greater than the MDC_90_ immediately post group. One other person with aphasia made a similar gain at 3‐month follow‐up. However, one participant demonstrated a negative change greater than MDC_90_ at follow‐up.

## 4 Discussion

Participants who attended the intensive conversation groups received a total of 26 h of Better Conversations CPT, delivered for 2.5–3 h per day, over 2 weeks (4 days a week). While delivered in a group, the intervention was designed to address individualised needs by supporting each dyad to set personal goals. All participants attended all sessions. Dosage was variably tolerated across the groups; PwPPA and their CPs reported the group was too long and intensive, while PwA and their CPs reported intensity and length were acceptable. Of 31 goals set, participants self‐rated as having achieved 26 goals and rated no change on five goals. Outcome measures indicated increased confidence (on CCRSA scores) and participation (on CPIB scores) across several participants with PPA and non‐progressive aphasia in both groups.

Participants were unanimous in their opinion that making connections with, and receiving support from, peers was highly valuable. This is consistent with best practice principles for speech and language therapy for PPA, which recommend ‘carefully plan opportunities for groups, and meeting others with a similar diagnosis’ to ‘connect people with a social network of people with shared experiences’ (Volkmer et al. 2023, p. 12). The finding also aligns with the work of researchers who have reported that meeting other dyads with PPA and aphasia enables people to share communication strategies, experience personal growth in psychological well‐being and renegotiate identity (Ross et al. 2006; Simmons‐Mackie et al. 2011; Volkmer et al. 2025). CPs of PwA in the current study made observations that PwA also benefited because they spoke more in the face‐to‐face group setting, as previously evidenced in stroke aphasia groups by Fama et al. 2016). Whilst the CPT intensive conversation groups enabled participants to build valuable relationships with others in the same situation, these groups were neither conversation groups nor support groups, but CPT delivered in a group, enabling the sharing of tips, hints and strategies and promoting generalisation of conversation behaviours. Remote sessions were generally dispreferred by participants in the current study, who highlighted a lack of group rapport and of opportunities for conversation practice in breaks, as compared to face‐to‐face sessions. Participants did however, recognise video conferencing as a vehicle for increased accessibility of therapy, and remote delivery of BC has been demonstrated to be acceptable to some individual dyads (Beeke et al. 2021). Importantly, participants did not suggest that groups should be delivered instead of individual therapy, but rather that groups provide a unique opportunity to meet others ‘in the same boat’. Participants expressed a desire for ongoing contact with each other after the end of the group and felt this was important for clinicians to facilitate for future groups.

PwPPA/PwA and their CPs consistently reported conversation strategies identified through goal setting to have improved their day‐to‐day interactions. Interestingly, while changes in mean Kagan Scale scores for PwPPA and their CPs corresponded with changes in conversation behaviours on goal setting, mean Kagan scores for PwA and their CPs did not change in the intended direction. There was also one dyad in the PPA group where both the PwPPA and CP scored at ceiling pre‐, post‐ and 3‐months post‐intervention, highlighting that the Kagan scales were not sensitive to proximal outcomes captured through self‐rating of goal attainment. Given the person‐centred goal setting reflected changes considered meaningful by participants, the lack of correspondence between the Kagan MPC/MSC scores and our goal achievement measure may suggest the Kagan measure is unable to capture meaningful change for intensive CPT groups. Comparatively, whilst time intensive, methodologies used to code and count conversation behaviours in video‐recorded conversations pre‐ and post‐intervention in previous BC intervention studies do align with goals set by participants in therapy (Best et al. 2016; Volkmer et al. 2023). This suggests that meaningful change cannot be captured by generic clinician‐rated scales, instead requiring close inspection of behaviours rated as important by participants themselves.

In this study, differences in disease trajectory (progressive versus non‐progressive) influenced the direction of outcomes. Confidence ratings improved immediately after the PPA group but were not maintained at 3‐month follow‐up. It is possible that disease progression may have contributed to the deterioration in confidence. Yet participation (on both CPIB and the Kagan MPC Scale) was maintained at 3‐months follow‐up. It is not possible to know what participant trajectories might have been without attending the intensive conversation groups. We might hypothesise a steeper decline across all measures over 3 months had individuals not participated in the intervention, meaning it is possible the intervention contributed to maintaining stability and slowing decline. Comparatively, confidence rose for the non‐progressive aphasia group and continued to rise at 3‐month follow‐up, with post‐intervention semi‐structured interview data suggesting a concurrent improvement in speaking for PwA (Theme 1 ‘His speaking obviously, has improved’ CP 2.03). Whilst more distal to the intervention, relationships improved for CPs across both groups immediately after group attendance. Relationship data (the QoCPR measure) was not collected at 3‐month review, due to participant feedback that the measure was not appropriate. It is possible that reflecting on relationships given the loss (in PPA) (Pozzebon et al. 2017) and the sometimes unfulfilled hopes for improvement (in non‐progressive aphasia) (Dar et al. 2025) cannot be captured by a rating scale (such as the QoCPR measure). Several researchers have highlighted the difficulty in measuring distal outcomes of CPT (Saldert et al. 2018), and whilst relationship outcomes suggest a change in the intended direction, future research should explore how best to capture this impact.

### 4.1 Limitations

Despite being a small service evaluation, this work is the first to explore dosage and intensity of CPT for non‐progressive aphasia and PPA. Importantly, the small sample size in the PPA group lacked representativeness, the PwPPA being monolingual English speakers from higher socioeconomic backgrounds. Several PwA declined to participate as their CPs were unable to attend due to barriers including a lack of time and financial means to pay for travel. While we tried to negate the financial burden by offering participants compensation for travel costs, and paying for accommodation and refreshments, it is possible that potential CPs from lower socioeconomic groups may not have the capacity to take annual leave to attend. These financial limitations may have broader feasibility implications for any intensive interventions such as those described in this study. Exploring barriers to uptake would inform implementation in future clinical contexts. Interestingly, none of the convenience sample participants recruited to the groups had a severe aphasia or semantic variant PPA, and sentence comprehension scores demonstrate relatively high performance (with the lowest score of 16/32 for one of the PwA). It is possible that this represents a bias in selection, and perhaps an assumption that participants required higher comprehension levels to participate in the group. Whilst PwA and PwPPA were excluded based on whether they had a serious mental health condition, CPs were not. Candidacy of both PwA or PPA and their CPs should be further explored in future CPT research. Importantly, the Friedman test used in this study was underpowered for PwA, with a minimum recommended sample size of 5, thus, statistical analysis should be interpreted cautiously. To negate the risk of overinterpretation, the MDC_90_ scores were used to explore individual participant changes, but given a lack of *t*‐test data for all measures, this was only applied for the CCRSA and CPIB. Measuring outcomes from CPT remains complex (Azios et al. 2022; Saldert et al. 2018), and moreover, measures are difficult to interpret in the context of a progressive disease trajectory. Whilst maintenance is an appropriate target, evidencing maintenance on tools designed for people with a non‐progressive condition may be limiting. Exploring qualitative feedback, as per Marshall et al. 2016) suggestion for complex speech and language interventions, may provide a more nuanced view of change.

### 4.2 Implications and Future Work

This service improvement project demonstrated that individuals living with PPA and non‐progressive aphasia and their CPs benefitted from CPT intensive conversation groups. Given the acceptability data, it is possible that adaptations could be better tailored to disease trajectory, such as a briefer intensive period for PwPPA, versus longer intensive groups for PwA and their CPs. Interestingly, the authors have successfully delivered group therapy less intensively (1‐h doses, once weekly) to PwPPA and PwA, suggesting that less frequent, short group doses delivered remotely may be better tolerated (Talbot et al. 2025). As the current project was a service improvement study, future research should make direct comparisons across different intensities, participant groups and severity to better inform our understanding of dosage benefits for CPT. The schedule (frequency) of speech and language therapy over the course of living with a condition has received little attention in the stroke aphasia literature to date (REhabilitation and Recovery of peopLE with Aphasia after StrokE (RELEASE) Collaborators 2022) but is recommended as a key consideration when working with PPA (Volkmer et al. 2023). Based on our experiences, we anticipate that PwPPA are more likely to benefit from intensive CPT interventions at an earlier disease stage with briefer, less intensive top ups over time. Whether this should be delivered in a group, and the schedule of intervention from diagnosis onwards requires further investigation to inform a care pathway. Similarly, we anticipate that further research on CPT for PwA may demonstrate benefits from bursts of intense intervention from acute into chronic stages. Future research on CPT dose needs to take account of the fact that individuals with non‐progressive and progressive conditions tolerate varying intensity of therapy. Importantly, CPT for non‐progressive and progressive aphasias should include group intervention options to enable people to share their experiences and strategies with others in the same position and facilitate enhanced practice opportunities.

## 5 Conclusion

This service improvement project demonstrates that CPT intensive conversation groups can be acceptable to participants with PwA, PwPPA and their CPs. Participants achieved their conversation goals and reported increased confidence. Participation improved or was maintained. Participants valued the group format and preferred face‐to‐face sessions, whilst intensity was variably tolerated. Future research is required to explore the benefits of different intensities of CPT and the schedule of interventions over different disease trajectories.

## Funding

AV is funded by National Institute for Health and Care Research (NIHR) Advanced Fellowship award (NIHR302240).

## Ethics Statement

This service improvement study was approved by the UCL Language and Cognition Ethics Committee, project ID: LCD‐2024‐05.

## Conflicts of Interest

The authors declare no conflicts of interest.

## Supporting information




**Supporting Information**: jlcd70296‐supp‐0001‐SuppMat.docx

## Data Availability

Data not available in the manuscript may be made available on request from the author.
