# Supplementary material for: Better Conversations Communication Partner Training for Progressive and Non‐Progressive Aphasia: What Can We Learn From Intensive Conversation Groups?
Source: Int J Lang Commun Disord. 2026 Jul 30;61(5):e70296. doi: 10.1111/1460-6984.70296 (PMC13425045; doi:10.1111/1460-6984.70296)
Supplement: Supplementary file 1 — Supporting Information: jlcd70296‐supp‐0001‐SuppMat.docx [file JLCD-61-0-s001.docx]

**Appendix 1: Topic guide for semi-structured interviews:**

Semi Structured Interviews

1. What was good about the group?
2. Is there anything that could be improved?
3. How do you feel about the intensity of the group?
4. How do you feel about the group format compared to individual therapy?
5. Do you have any feedback about Zoom sessions compared to in-person?
6. Has there been any changes in your conversations, if so, what?
7. What is the most useful thing you will take away from the group?
8. If you were inviting someone else to the group, what would you say?

3-month post group questions

1. Have there been any lasting changes in your conversations and if so what are they?

2. As it is now 3-months after the group, do you have any additional comments about the group? (e.g. what was useful, what could have been changed, what else do you need now)

**Appendix 2: Detailed description of assessment and outcome measurement tools**

| Measurement tool | Description of the measurement tool |
| --- | --- |
| Sentence Comprehension and picture description task from the Comprehensive Aphasia Test (CAT)  *Completed only by PwA or PwPPA.*  *Scored by student SLTs (LD & KW), masked to time point of recording.* | On the sentence comprehension task participants were given a sentence (verbally) and asked to select a picture from a choice of four that best matched the sentence. In line with the CAT instructions, they were given one practice item and 16 test items. They were scored for accuracy and promptness on each item.  Participants were also video recorded providing a verbal description of a composite picture. Recordings were scored in line with the CAT manual by recording the total number of appropriate Information Carrying Words (ICWs), and deleting the number of inappropriate ICWs, syntactic variety 0-6 (0= no syntactic structure or stereotyped use of syntactic structure; 6= a full or nearly full range of structures used), grammatical well formedness 0-6 (0= No phrases well formed; 6= all phrases well formed; no phrases omitted) and the speed of speech production 0-3 (0= Significant and consistent delay; 3= Normal speed of delivery). |
| Kagan Scales: Measure of Skill in Supported Conversation (MSC) and Measure of Participation in Conversation (MPC) (Kagan et al., 2018)  *Scored by LD and KW, masked to time point of recording.* | Video recordings of conversations are by a naïve assessor using the Kagan scale (ref) across domains on a scale from 0-4 (0 = lower score, 4 = excellent score). Domains rated for the PwPPA or PwA were rated on i*nteraction* and *transaction* whereas the CPs were rated on *revealing competence* and *acknowledging competence.* |
| Goal Achievement | Participants set goals to work on during the first day of the intensive groups. Participants were then asked to rate the importance (as important, or less important) and achievability (more or less achievable) of their goals. At the end of the intervention period, participants were asked to rate their goals as no change, achieved or overachieved. |
| Communication Confidence Rating Scale for Aphasia (CCRSA) (Babbit et al, 2011)  *Completed only by PwA or PwPPA* | A ten-item rating scale asking participants to rate their confidence in various communication situations on a scale from 0-100 (100= very confident, 0= not confident) |
| Communication Participation Item Bank (CPIB) (Baylor et al, 2013)  *Completed only by PwA or PwPPA* | A ten-item rating scale asking participants to rate how much their communication difficulties interfere with communication participation on a scale from 0-3 (0=very much, , 3=not at all) |
| Quality of Caregiver Patient Relationship (QoCPR)  *Completed by both members of the dyad* | A 14-item rating scale asking participants to rate agreement/disagreement with relationship-related statements such as “I enjoy spending time with my partner” |

**Appendix 3:** **TIDieR (Template for Intervention Description and Replication) Checklist for Better Conversations**

| Item Number | Item |
| --- | --- |
| 1. | **Brief name**  Better Conversations (BC) |
| 2. | **Why**  BC is a manualised communication partner training intervention. The goal of BC is to improve communication between people with communication difficulties (such as stroke related aphasia, primary progressive aphasia and Parkinson’s) and their conversation partners (CPs), who are both involved in the training. This intervention was initially developed for people with stroke aphasia (Better Conversations with Aphasia - BCA) and has been refined to meet the needs of people with Primary Progressive Aphasia (Better Conversations with PPA - BCPPA) and Parkinsons (Better Conversations with Parkinsons - BCP). The rationale is that by working with people with communication difficulties and their partners, use of t jointly negotiated communication strategies will be improved and maintained. BC is based on applied Conversation Analysis and behaviour change theory [1]. BC provides multiple opportunities for people to reflect on and practice strategies in therapy sessions, using video feedback to support participants to reflect on the impact of their communication on one another, with a focus on elimination of barriers as well as implementation of facilitator strategies. BC emphasises the joint setting of goals during therapy, facilitated by the speech and language therapist (SLT), thus implementing principles of self-management and self-efficacy [2] to set achievable and personally relevant goals. |
| 3. | **What**  Materials:  The BC programs are accompanied by session plans, to guide the SLT, outlining specific goals and activities for the sessions. Participants pre-intervention video-recorded conversation samples are used to provide clips for video feedback during intervention sessions. Handouts and homework sheets have been designed to accompany each session should they be required. The BC team provide training to researchers and SLT collaborators in all their studies. The BC Lab offers the training externally twice annually. Some of the BC resources can be accessed by creating a free account and logging into the BCA training on the UCLeXtend website at <https://extendstore.ucl.ac.uk/product?catalog=uclxbca> and on the BC Lab website (<https://www.ucl.ac.uk/brain-sciences/pals/research/language-and-cognition/language-and-cognition-research/better-conversations-lab/better-conversations-resources-and-downloads>) and are publicly accessible. |
| 4. | **What**  Procedures: The person with the communication difficulties and their CP (a dyad) are provided with information and education on conversation (what it is and how it works). The dyad is then supported to reflect on video samples of their own conversation to identify behaviours that facilitate or are a barrier to communication. Consequently, participants are supported to set goals using our goal achievement measure to identify target communication strategies which they practise during activities, role play and homework tasks. Finally, the dyad is supported to plan for future communication needs such as sharing strategies with friends. |
| 5. | **Who provided**  In the BC research studies BC is delivered by qualified speech and language therapists, who have been trained using the online training modules accompanying the intervention program. |
| 6. | **How**  BC is delivered face to face or online via video conferencing to a person with communication difficulties and their chosen CP. BC may also be delivered in group sessions with other dyads present. Whether online or in person we typically recommend no more than 4-5 dyads in a BC group. |
| 7. | **Where**  BC can be delivered at home or in an outpatient type-setting, depending on the location of speech and language therapy provision. It can also be delivered remote via video conferencing. |
| 8. | **When and how much**  The BC programs are delivered based on the requirements of the dyad and how long it takes them to achieve their goals. However constraints such as the available services may influence this decision. Typically, individual BC programs are provided once weekly for around 60 minutes. Group BC programs may be delivered over shorter and more intensive time frames. Individual BC intervention research will provide detailed information about number and duration of sessions, schedule and frequency. |
| 9. | **Tailoring**  BC is tailored to each individual dyad (a person with communication difficulties and their CP) by using video samples of their conversation recorded before intervention. These samples permit the SLT to assess the dyad’s communication difficulties and strengths before commencing the program. They are also used for video feedback to allow a dyad to set goals i.e. target communication strategies, which they practise during activities, role play and homework tasks. |
| 10. | **Modifications**  BC interventions are modified for each intervention study. For the current study the dosage and intensity of BC were modified to be delivered in 26 hours over eight days (see Table 2 in the manuscript). Core components described in item 4 of the current TiDIER were augmented with a range of practice activities such as dialogic art activities, group conversations, creative activities, guided walks and discussion around how to share strategies with other people. |
| 11. | **How well**  Typically, BC intervention are video recorded for fidelity assessment and a detailed protocol has been developed to support the BC program of research [3]. Given this service improvement project was not aiming to explore fidelity, this was not explored in this study. |

1. Johnson FM, Best W, Beckley FC, Maxim J, Beeke S. Identifying mechanisms of change in a conversation therapy for aphasia using behaviour change theory and qualitative methods. Int J Lang Commun Disord. 2017;52:374–87. doi:10.1111/1460-6984.12279.

2. Yorkston K, Baylor C, Britton D. Incorporating the Principles of Self- Management into Treatment of Dysarthria Associated with Parkinson ’ s Disease. Semin Speech Lang. 2017;1:210–9.

3. Volkmer, A., Beeke, S., Warren, J. D., Spector, A., & Walton, H. (2024). Development of fidelity of delivery and enactment measures for interventions in communication disorders. *British Journal of Health Psychology*, *29*(1), 112-133.

Appendix 4. *Individual Participant Outcomes*

*= The Minimal Detectable Change (MDC_90_), defined as the minimal score change that falls outside measurement error with 90% confidence, was used to identify changes in individual participants' confidence, as measured by the CCRSA (≥ 5.96), consistent with methodology applied by Dignam et al (2025). Similarly, the MDC_90_ for the CPIB (≥4.61), as applied by Carlozzi et al (2021), was used to identify changes in individual participants’ participation.

|  |  | Communication Confidence Rating Scale in Aphasia (CCRSA) | | |
| --- | --- | --- | --- | --- |
|  | Participant ID | Pre-Intensive Group | Post-Intensive Group | 3-month follow up |
| PPA Group | 1.01 | 75 | 71 | 69* |
|  | 1.02 | 60 | 51* | 51 |
|  | 1.03 | 64 | 72* | 64* |
|  | 1.04 | 55 | 65* | 48* |
|  | 1.05 | 48 | 53 | 39* |
|  |  |  |  |  |
| Non Progressive Aphasia Group | 2.01 | 36 | 40 | 47* |
|  | 2.02 | 74 | 71 |  |
|  | 2.03 | 53 | 56 | 71* |
|  | 2.04 | 32 | 34 | 45* |

|  |  | Communication Participation Item Bank (CPIB) | | |
| --- | --- | --- | --- | --- |
|  | Participant ID | Pre-Intensive Group | Post-Intensive Group | 3-month follow up |
| PPA Group | 1.01 | 5 | 5 | 7 |
|  | 1.02 | 13 | 14 | 19* |
|  | 1.03 | 10 | 11 | 12 |
|  | 1.04 | 10 | 7 | 8 |
|  | 1.05 | 6 | 6 | 7 |
|  |  |  |  |  |
| Non Progressive Aphasia Group | 2.01 | 0 | 2 | 9* |
|  | 2.02 | 10 | 15* |  |
|  | 2.03 | 18 | 15 | 13* |
|  | 2.04 | 0 | 2 | 3 |
